# Genetic Surveillance Detects Both Clonal and Epidemic Transmission of Malaria following Enhanced Intervention in Senegal

**DOI:** 10.1371/journal.pone.0060780

**Published:** 2013-04-04

**Authors:** Rachel Daniels, Hsiao-Han Chang, Papa Diogoye Séne, Danny C. Park, Daniel E. Neafsey, Stephen F. Schaffner, Elizabeth J. Hamilton, Amanda K. Lukens, Daria Van Tyne, Souleymane Mboup, Pardis C. Sabeti, Daouda Ndiaye, Dyann F. Wirth, Daniel L. Hartl, Sarah K. Volkman

**Affiliations:** 1 Department of Immunology and Infectious Diseases, Harvard School of Public Health, Boston, Massachusetts, United States of America; 2 Broad Institute, Cambridge, Massachusetts, United States of America; 3 Department of Organismic and Evolutionary Biology, Harvard University, Cambridge, Massachusetts, United States of America; 4 Faculty of Medicine and Pharmacy, Cheikh Anta Diop University, Dakar, Senegal; 5 Department of Human Evolutionary Biology, Harvard University, Cambridge, Massachusetts, United States of America; 6 FAS Center for Systems Biology, Harvard University, Cambridge, Massachusetts, United States of America; 7 School of Nursing and Health Sciences, Simmons College, Boston, Massachusetts, United States of America; Barcelona Centre for International Health Research/Hospital Clinic/IDIBAPS/University of Barcelona, Spain

## Abstract

Using parasite genotyping tools, we screened patients with mild uncomplicated malaria seeking treatment at a clinic in Thiès, Senegal, from 2006 to 2011. We identified a growing frequency of infections caused by genetically identical parasite strains, coincident with increased deployment of malaria control interventions and decreased malaria deaths. Parasite genotypes in some cases persisted clonally across dry seasons. The increase in frequency of genetically identical parasite strains corresponded with decrease in the probability of multiple infections. Further, these observations support evidence of both clonal and epidemic population structures. These data provide the first evidence of a temporal correlation between the appearance of identical parasite types and increased malaria control efforts in Africa, which here included distribution of insecticide treated nets (ITNs), use of rapid diagnostic tests (RDTs) for malaria detection, and deployment of artemisinin combination therapy (ACT). Our results imply that genetic surveillance can be used to evaluate the effectiveness of disease control strategies and assist a rational global malaria eradication campaign.

## Introduction

The *Plasmodium falciparum* malaria parasite causes nearly 700,000 deaths annually, primarily in sub-Saharan Africa [1], where disease prevalence and transmission intensity are highest. Because parasite populations are large in Africa, they are more genetically diverse there than elsewhere. They also exhibit less correlation between allelic states at different loci (i.e. less linkage disequilibrium, or LD), reflecting both the large population and also higher disease transmission rates, which facilitate sexual outcrossing [2]–[6].

We sought to use changes in parasite population diversity to detect longitudinal changes in disease transmission, and thereby to develop useful metrics for monitoring antimalarial interventions. As a tool to track parasite diversity, we employed a previously developed ‘molecular barcode’, composed of assays for 24 single nucleotide polymorphisms (SNPs) across *the P. falciparum* genome [7]. We applied the barcode to samples from Senegal. Since 2005, Senegal has dramatically increased deployment of intervention strategies, including ITNs for prevention, RDTs for detection, and ACTs for treatment, resulting in an overall decline in a number of malaria indicators [8], and making it a good site for detecting changes in parasite diversity.

## Results

### Identification of repeated barcodes

**Figure 1:**
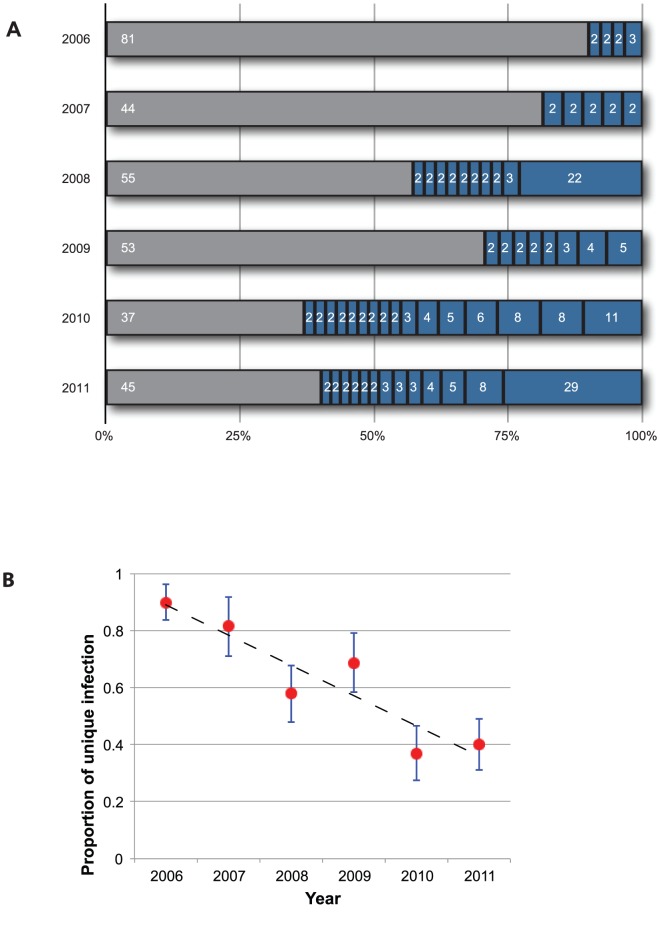
Temporal changes in population characteristics. **A.** **Decreasing prevalence of unique parasite barcode profiles.** For every collection season, the number of samples with unique barcodes (grey) and the number of samples in each shared-barcode cluster (blue) are shown. **B.** **Ratio of shared vs. unique barcode profiles.** The proportion of samples residing outside of shared-barcode clusters is shown per year. The error bars show 95% confidence interval of mean (±1.96 SE).

We sampled patients annually from 2006–2011, from the Service de Lutte Anti-Parasitaire (SLAP) clinic in Thiès, Senegal under ethical approval, and genotyped the samples using the barcode (Methods). We first compared molecular barcodes within and between years. We confined this analysis to infections caused by a single parasite strain to reduce ambiguity from heterozygosity. The most prominent signal in our longitudinal collection of molecular barcode data was a steady increase in the number of identical barcodes observed in distinct patient samples (Figure 1A). Whereas 10% of samples shared barcodes during the 2006 transmission season, more than 50% were within identical-barcode clusters in 2010 and 2011. Repeated instances of the same barcode were not limited to clusters of 2 or 3; in 2008 one barcode was observed in 22 distinct patient samples, and in 2011 nearly a quarter of the sampled infections exhibited another shared barcode. Overall, the proportion of unique parasite types decreased significantly over the study period (Figure 1B; *P* = 0.006, ANOVA). We investigated whether parasite samples exhibiting identical SNP barcodes are also genetically identical at other sites in the genome by hybridizing multiple clusters of samples with shared barcodes to a whole-genome SNP array that interrogates 17,000 polymorphic positions [6]. Parasite samples sharing barcodes exhibited array-based genotype profiles as similar to each other as technical replicate hybridizations of a single laboratory reference strain (Figure S1), suggesting that samples sharing barcodes are nearly genetically identical and likely derived from the same ancestor.

### Clonal propagation vs. epidemic expansion

**Figure 2:**
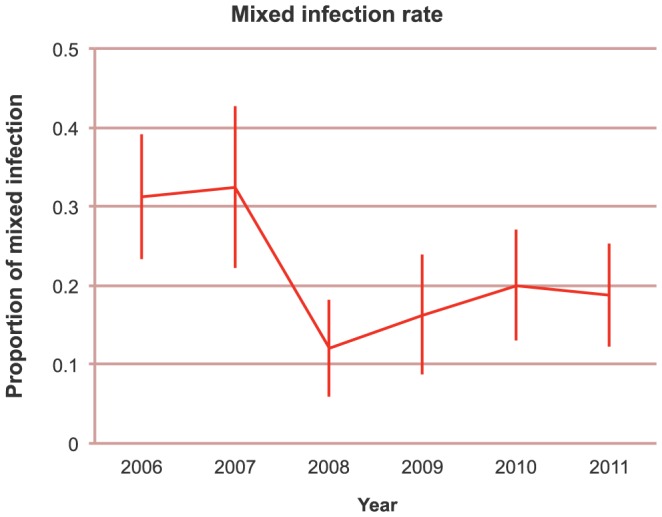
Mixedness over time. Proportion of mixed infections decreased between 2007 and 2008. The error bars show 95% confidence interval of mean (±1.96 SE).

The increasing occurrence of repeated barcodes (i.e. nearly genetically identical samples) in later years could be attributed to either “clonal propagation” or “epidemic expansion”, or both. Clonal propagation is intrinsically linked to low parasite transmission, owing to the life history of *Anopheles* mosquito vectors. Female *Anopheles* mosquitoes ingest haploid *P. falciparum* gametocytes during a blood meal from a human host. The gametocytes differentiate into gametes in the mosquito midgut, where they unite to form a diploid zygote, which in turn undergoes meiosis to restore haploidy prior to inoculation of the next human host. Genetic outcrossing during the parasite’s sexual stage occurs only when a mosquito bites a host infected simultaneously by multiple parasite strains and gametocytes from multiple genetically distinct strains circulate in the blood of a host; bites of singly-infected hosts result in the union of nearly genetically identical gametes in the mosquito midgut, and consequently result in self-fertilization and clonal parasite transmission. To test this possibility, we compared the proportion of multiple infections over time and found that the proportion of mixed infections was significantly greater in 2006–2007 compared to subsequent years (Figure 2 and Table S1). While the patient parasitemia reported for those years varied between years, there was no trend in decreased parasitemia or sampling bias that could contribute to the trend (Figure S2). This pattern of decreasing proportion of multiple infections is consistent with the decrease in the proportion of unique barcodes in Figure 1B, suggesting that “clonal propagation” due to decreased outcrossing is also consistent with the appearance and increase of repeated barcodes.

**Figure 3:**
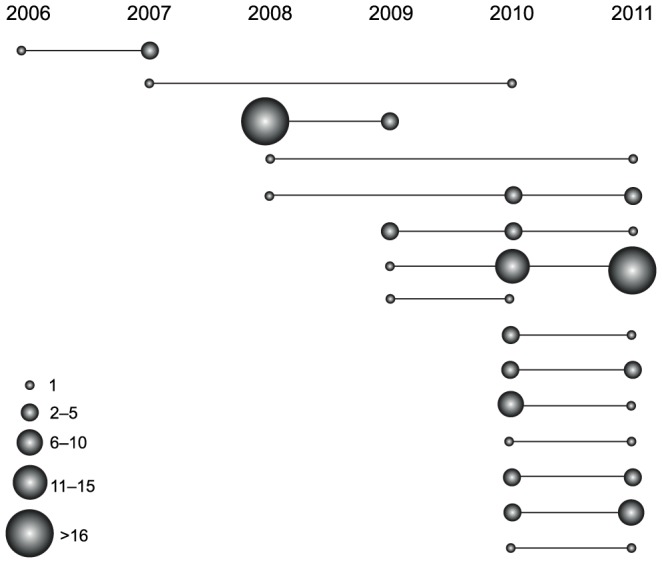
Clonal transmission of parasites across transmission seasons. Size and distribution of same parasite types across collection years.

“Epidemic expansion” means that particular clones expand in the population, perhaps due to advantageous haplotypes, or a founder effect at the beginning of each transmission season, or both. Factors promoting variance in reproductive success, such as enhanced production of gametocytes, evasion of the host immune response, or enhanced transmission by selected or alternative mosquito vectors could select and enrich for favored parasite lineages in the population. Epidemic expansion is supported by the observation of two exceptionally prevalent barcodes in 2008 and 2011 (shown in Figure 3). To further test the possibility of epidemic expansion in our population, we used the framework described in Maynard Smith et al. [9] and Anderson et al. [4]. We compared multilocus linkage disequilibrium (LD) using the standardized index of association (*I_A_^S^*) [10], when including and excluding samples with the same barcode. The result shows significant LD from 2008 to 2011 when all samples are included, and no significant LD when only considering unique barcodes (Table 1), suggesting that the significant LD from 2008 to 2011 is caused by repeated barcodes; that is, some epidemic clones. There is no significant LD in 2006 and 2007 whether we included or omitted repeated barcodes. The lack of significant LD in 2006 and 2007, and the restoration of linkage equilibrium from 2008 to 2011 after excluding repeated barcodes suggest that the background population is still under linkage equilibrium and the decrease in the population recombination rate due to lowered transmission is very recent. Taken together with our analyses of the proportion of mixed infections, a likely explanation for these observations is a reduction of outcrossing in 2007–2008 followed by an expansion of individual parasite genotypes.

**Table 1:** Multilocus linkage disequilibrium.

		2006	2007	2008	2009	2010	2011
All samples	I_A_^S^	−0.0049	0.0049	0.0306	0.0072	0.0293	0.1111
	*P*-value	0.989	0.162	<1×10^−4^	0.021	<1×10^−4^	<1×10^−4^
Unique barcodes only	I_A_^S^	−0.0059	0.002	0.0012	0.0038	0.0042	0.0041
	*P*-value	0.997	0.332	0.329	0.151	0.145	0.128

Moreover, we examined whether parasite samples with shared barcodes were collected in proximal dates. The difference in collection dates among samples with identical barcodes is significantly smaller than that among samples with different barcodes (Wilcoxon rank sum test, *P* = 0.005), suggesting temporal expansion of particular clones in the population. However, because there was no temporal trend of increasing prevalence of a single parasite type (Figure 3) we do not believe that this was a selection event caused by emergence of drug resistance. It could possibly be a selection caused by emergence of resistance to host immune response, but the advantage disappears over time due to the corresponding changes in host, or non-selective forces. Alternatively, it might be possible that the parasite clones that appear to expand in the community were derived from an imported line novel to the area and thus the local population has little "strain-specific" immunity. We compared the pairwise differences between two exceptionally prevalent barcodes in 2008 and 2011 and the rest of strains with the pairwise differences among all strains from the same year, and found that the differences between two prevalent repeated barcodes and the rest of strains are not significantly higher than the differences among all strains from the same year (Figure S3). This result indicates that we do not observe the evidence of imported lines from the current data. Additional sequence information of polymorphic sites will be helpful to distinguish migrants from local population.

### Effective population size

Reduced transmission can lead to lower parasite effective population size (*N_e_*). To test whether the deployment of intervention strategies in recent years reduces malaria transmission, we examined the parasite effective population size (*N_e_*). Population genetic theory predicts that a decreasing population should undergo increased genetic drift, manifested as increasingly variable allele frequencies across generations. The relevant measure of effective size in this context is the variance effective population size; this we estimated by measuring the fluctuation in allele frequencies across transmission seasons of the SNPs comprising the molecular barcode. We observed large fluctuations in allele frequencies over time (Figure S4). The variance *N_e_* was calculated by all polymorphic SNPs using a likelihood approximation (Methods) and observed an extremely small variance *N_e_* over time (Table 2 and Table S2). The estimated variance effective size in 2011 is only 10, a strikingly low value that reflects large fluctuations in allele frequencies. In order to exclude the possibility that some particular parasite types are so successful in the population that lower the estimate of effective population size, we also calculated *N_e_* by counting each repeated barcode once (Table S2). The estimates of *N_e_* are still very small (less than 250) although some of the confidence intervals could not be determined. This extremely small effective population size predicts low effectiveness of selection efficiency and low rate of adaptation in Senegal.

**Table 2:** Variance effective population size estimated by likelihood approximation.

	Likelihood method	
	Mean	95% Confidence Interval
2006–2007	ND	(226, ND)
2007–2008	19	(9, 49)
2008–2009	29	(12, 90)
2009–2010	18	(9, 42)
2010–2011	10	(6, 18)

* ND represents “Not Determinable”.

### Persistence across years

We also investigated the barcode dataset for evidence of clonal parasite persistence across years. Malaria transmission in Senegal is sharply seasonal, coinciding with annual rainfall patterns. Some parasite clones did indeed appear in more than one transmission season (Figure 3). These included clonal parasite types that persisted into the subsequent year and some that persisted longer, sometimes reappearing two or three seasons after initial detection. The increasing ratio of parasites persisting between years from 2006 to 2011 was statistically significant (*P = *0.008, ANOVA) (Figure S5). Notably, we found an increase in the frequency of identical-barcode parasites persisting between 2010 and 2011: of the 15 identical barcodes that persisted for at least one year, ten were found during that pair of years. Because parasite samples sharing the same barcode are likely to be identical by descent, the persistence of identical barcodes across years suggests multiple sequential transmission cycles among singly-infected hosts, and indicates clonal propagation.

To explore the patterns of repeated barcodes, and to rule out sampling biases in our study design, we examined the spatial and temporal relationships between samples exhibiting identical barcodes. We insured that clonal parasites were derived from independent natural infections by assaying 18 SNPs in the human host genetic material. We found no evidence of serial sampling of the same host among samples exhibiting the same barcode (Table S3). Examination of patient data confirmed that barcodes observed more than once were not clustered by household, ruling out a simple hypothesis of transmission among family members. Further analysis of the parasites within these samples by sequencing of the highly-polymorphic T-epitope region of the *csp* gene provided further evidence of highly related parasites (Table S4). We found that samples with identical barcodes are distributed across the entire transmission season and clinical catchment area, indicating a lack of temporal or spatial clustering. Our data therefore suggest a regional-level change in transmission dynamics from 2006 to 2011, rather than localized shifts.

Moreover, we compared ages of hosts before and after we observed the significant increase in the frequency of repeated barcodes. There is no significant difference in host ages between 2006–2007 and 2008–2011 (t test, *P* = 0.094), suggesting that the patterns of identical barcodes are unlikely to be confounded by host ages.

## Conclusions and Discussion

With the restructuring of the National Malaria Control Programme (NMCP) in 2005, Senegal implemented an organized approach to malaria control and elimination. From 2006 to 2010, the NMCP increased access to insecticide-treated bednets (ITNs) and residual insecticide spraying, with the number of reported bednets per home increasing more than 35% from 2008 to 2010. Combined with no-charge access to ACTs from 2007, the country reported a 41% drop in the number of malaria cases between 2008 and 2009 [8]. The findings of increasing repeated barcodes, persistence, and proportion of single infections across transmission seasons demonstrate the usefulness of genetic tools for monitoring the effectiveness of intervention strategies against infectious disease. This type of evidence could inform control efforts as a real-time gauge of the progress towards control, elimination, or eradication. Our ability to differentiate between clonal and epidemic population structures and to track these changes within the population could lend a more refined view of the subtle effects and varying degrees of effectiveness in control programs.

While our study reported the first evidence of clonal propagation and epidemic expansion in Africa, other groups have also used genetic tools to study parasite dynamics in geographically distinct regions, and reported clonal lineages and persistence over time [11]–[13]. Roper et al. showed the persistence of parasites over the dry season in Sudan and Echeverry et al. showed similar in Colombia [14], [15]. Both Branch et al and Griffing et al point to distinct genetic types within South America and Peru in particular, and attribute population patterns to periodic epidemics in regions with relatively low transmission levels [11], [12]. Similarly, Nkhoma et al showed the decreases in the proportion of unique parasite genotypes and the proportion of multiple infections along with large reduction in transmission over time. However, they found no evidence of reduction in *N_e_* during the same period of time, which was possibly caused by migrations between nearby populations, or the lack of power in analysis of temporal data when the true *N_e_* is not small enough [13]. Moreover, Mobegi et al. 2012 showed that the background of non-clonal population structure has been widespread elsewhere surrounding our study area in West Africa, indicating that there has been dramatic changes in the population structure of this site in contrast to the surrounding regional parasite population structure [16]. These studies, including our study, indicate the power of using genetic tools to study parasite population structure, and highlight the need for further detailed study of parasite population dynamics in more extensive geographical regions to understand the interactions and migrations between different parasite populations.

Further applications of this approach might be to differentiate between parasite recrudescence or re-emergence in selected populations to allow facile decision-making in the face of a very changeable parasite where resistance emerges quickly[7]. With additional evidence provided by other types epidemiological studies to more directly link these parameters to parasite population genetics, changes in the profile of parasites with different molecular barcodes might be used as an indicator of parasite transmission. The finding is also one beneficial outcome of a genomic diversity project undertaken by the malaria community five years ago [2], [17], [18]. The decreasing cost and increasing translation of sequencing and genotyping tools into clinical environments will make genetic data invaluable for rapidly understanding diverse aspects of infectious disease epidemiology, particularly when such information is combined with population genetic inferences and knowledge of pathogen biology.

## Materials and Methods

### Study site

We obtained *P. falciparum*-positive clinical samples from patients evaluated at the SLAP clinic in Thiès, Senegal under ethical approval for human subjects and informed consent conditions. Full written consent was obtained in a protocol approved by Harvard School of Public Health, Office of Human Research Administration (P16330-110, Wirth PI) and the Ministry of Health, Senegal.

The site, located 75 km southeast of the country capital of Dakar, is characterized by perennial hypo-endemic transmission with the greatest number of malaria cases by primarily *Anopheles gambiae s.l* and *A. funestus* vectors occurring approximately from September to December, at the end of the rainy season. Samples are collected passively; with patients over the age of 12 months admitted to this study with self-reported acute fevers within 24 hours of visiting the clinic and no recent anti-malarial use. Patients are screened by slide smears and rapid diagnostic test (RDT) to diagnose *P. falciparum* infection [19], [20].

### DNA extraction and quantification

Whole blood spots from 2006–2011 were preserved on Whatman FTA filter paper (Whatman catalog #WB120205). We extracted genomic DNA from 4–6 mm punches from the FTA cards using the manufacturer protocol for Promega Maxwell DNA IQ Casework Sample kit (Promega catalog #AS1210). After extraction, we quantified and generated a molecular barcode for each sample as described previously [7]. Extracted samples were excluded from analysis if the concentration (and corresponding parasitemia of the patient) were too low for successful amplification. The sample size in each year is shown in Table S1.

### Sequencing csp

We sequenced across the T-epitope region of the *P. falciparum csp* gene. Primer sequences were: 5’- AAATGACCCAAACCGAAATG-3’ forward and 5’- TTAAGGAACAAGAAGGATAATACCA-3’ reverse. We used 1 µl of each sample as a template in 25 µl PCR reactions using iProof master mix (Bio-Rad cat# 172-5310) (initial denaturation 98°C 30 s, followed by 35 cycles of 98°C denaturation (30 s), 55°C annealing (30 s), 72°C extension (30 s), and a final extension of 72°C for 5 min) and sent post-PCR processed samples (exoSAP-IT, usb catalog #78201) for sequencing (Genewiz, Inc., South Plainfield, NJ).

### Affymetrix array analysis

Using an Affymetrix array containing 74,656 markers [6], we hybridized parasites with identical barcodes and parasites within the same collection but with different barcodes as well as technical replicates of control strains. We called SNPs using BRLMM-P from Affy Power Tools v1.10.2.Haploid genotypes were forced by designating all SNPs as "Y chromosome" and all individuals as "male". We counted the number of differing SNP genotypes for pairs of arrays, with pairings sorted into three categories: 1) technical replicates (same parasite sample hybridized to two arrays); 2) identical barcodes (distinct patient samples with identical barcodes); and, 3) unrelated parasites (distinct barcodes).

### Human Genotyping

We used a set of SNPs selected by The Broad Institute for human typing on their analysis platforms to distinguish patient samples from one another. From an original set of 23 assays, we selected 18 as robust under conditions with low template concentrations. We ran these pre-developed TaqMan-MGB probes (Life Technologies, Inc.) on an Applied Biosystems 7900HT qrt-PCR system (LifeTechnologies, Inc.) using the standard amplification and analysis protocols (see Table S3A for SNP identity and human typing results).

In addition, we sent several samples for STR genotyping on an ABI 3130 Genetic Analyzer to detect the STR alleles amplified using the ABI AmpFlSTR Profiler Plus Kits (Life Technologies catalog # 4303326) at the Histocompatibility and Tissue Typing Laboratory, Brigham and Women's Hospital, Boston, MA. See Table S3B for results of this genotyping.

### Data Analysis

We excluded from analysis those samples with missing data on more than four SNP positions. We determined that samples with more than one site showing both fluorescent signals in genotyping (indicating that more than one allele were present) were “mixed infections” with more than one genome present in the patient sample. For simplicity, the results we show in the paper are all based on samples with single genome. We also considered mixed infection in the analyses, and the results do not change qualitatively.

We calculated the standardized index of association (*I_A_^S^*) by the program LIAN, version 3.5 [10]. The number of re-samplings was set to be 10,000. We assumed there are two generations per year and estimated variance effective population size through temporal changes in allele frequencies by both the moment method [21] and likelihood approximation implemented in program CoNe [22]. We calculated the ratio of parasites persisting between years in each year through dividing the number of barcodes that are shared with other years by the total number of barcodes in a particular year.

## Supporting Information

Figure S1 The percent differences between hybridized biological replicates and samples with identical barcodes. Array analysis shows that the percentage of SNP differences between samples with identical barcodes is similar to those seen in biological replicates, suggesting that samples with identical barcodes are nearly genetically identical.
(EPS)

Figure S2 Parasitemia variation by year. Kruskal-Wallis rank-sum test indicates variance between years (p-value < 2.2e-16); however, there is no decreasing trend over time.
(EPS)

Figure S3 Pairwise differences between two exceptionally prevalent barcodes and other barcodes. The number of pairwise differences between the exceptionally prevalent barcode and other barcodes is not significantly higher than the number of pairwise differences among all the barcodes in the same year.
(EPS)

Figure S4 Changes in allele frequencies between transmission seasons. Each colored line shows the year-to-year variability in allele frequency for each non-fixed SNP. Each colored line shows the year-to-year variability in allele frequency for each non-fixed SNP. Allele frequencies fluctuate substantially across years, suggesting high random genetic drift and low effective population size.
(EPS)

Figure S5 Proportion of between-year shared barcodes. Proportion of between-year shared barcodes increased significantly. The error bars show 95% confidence interval of mean (±1.96 SE).
(EPS)

Table S1 The number of mixed and single infections in each year.
(XLSX)

Table S2 Variance effective population size.
(XLSX)

Table S3 Human genotyping data. A.TaqMan probes from Broad Institute set. B. STR genotyping on an ABI 3130 Genetic Analyzer
(XLSX)

Table S4 csp sequences.
(XLSX)

Table S5 Complete molecular barcodes for all samples studied. SNP called marked ‘-‘ indicate missing data
(XLSX)
